# The impact of body composition and systemic inflammatory markers on postoperative complications in early-stage cervical cancer

**DOI:** 10.3389/fonc.2025.1696383

**Published:** 2026-01-06

**Authors:** Lipeng Ding, Shuangxi Li, Zhuanmei Jin, Changyu Yao, Taohua Zhang, Wenzhen Yuan

**Affiliations:** 1 The First Clinical Medical College of Lanzhou University, Lanzhou, China; 2 Department of Radiation Oncology, Gansu Provincial Maternity and Child-care Health Hospital, Lanzhou, China; 3 The Department of Oncology, The First Hospital of Lanzhou University, Lanzhou, China

**Keywords:** body composition, cervical cancer, postoperative complications, sarcopenia, systemic inflammation

## Abstract

**Objective:**

Patients with cancer often present with alterations in body composition and systemic inflammation. Guided by this observation, we sought to determine whether these factors are associated with postoperative complications in early-stage cervical cancer.

**Methods:**

In this retrospective cohort study, we analyzed data from 223 patients with early-stage cervical cancer treated at our center between July 2018 and December 2021.Postoperative complications were graded using the Clavien-Dindo classification. Systemic inflammatory markers were calculated from hematological parameters, while body composition indices were derived from computed tomography (CT) images at the third lumbar vertebra (L3) level and patient’s height. Group comparisons were performed using the χ² test, independent samples t-test, or Mann-Whitney U test, as appropriate. Binary logistic regression was applied to identify independent predictors of complications and conduct to sensitivity analysis to verify the robustness of the model.

**Results:**

Preoperative CT scans and hematological tests were performed a mean of 3.4 and 4.1 days before surgery, respectively. Postoperative complications were predominantly Grade 1 (n=124, 55.6%), followed by Grade 2 (n=69, 30.9%) and Grade 3 (n=30, 13.5%). No complications ≥ Grade 4 were observed. Patients with complications ≥ Grade 2 were more likely to undergo open surgery, present with larger tumors, exhibit lower skeletal muscle index (SMI) and prognostic nutritional index (PNI), higher visceral adipose tissue index (VATI), as well as greater intraoperative blood loss, lower preoperative and postoperative albumin levels, increased thrombotic risk, and longer hospital stays. Multivariate analysis identified that a higher complication grade (≥Grade 2) was significantly associated with low SMI (OR = 0.34, 95% CI = 0.18–0.64, P < 0.001), high VATI (OR = 1.04, 95% CI = 1.02–1.06, P < 0.001), and low PNI (OR = 0.91, 95% CI = 0.84–0.98, P = 0.008). No significant associations were found with IMATI, SATI, or systemic inflammatory markers (NLR, PLR, LMR, NPR, SII). After conducting sensitivity analyses on the key findings, the results remained robust.

**Conclusion:**

Sarcopenia, visceral adiposity, and low PNI are independently associated with a higher risk of clinically significant postoperative complications in patients with early-stage cervical cancer.

## 1 Introduction

Cervical cancer ranks fourth worldwide in both incidence and mortality among female cancers (1). Its burden is unevenly distributed, with approximately 85% of cases in low- and middle-income countries (2). In China, the incidence and mortality rates remain higher than in the United States and other Western countries (3). Treatment strategies vary by stage: radical hysterectomy is standard for early-stage disease, radical concurrent chemoradiotherapy for locally advanced disease, and systemic therapy for advanced or metastatic disease (4). For patients with early-stage cervical cancer (FIGO 2018 stage ≥ IB1), Querleu-Morrow type C radical hysterectomy is recommended. However, due to its extensive surgical scope and prolonged operative time, the incidence of severe postoperative complications (≥ G3) ranges from 10.1% to 25.4% (5).

In recent years, with the continuous advancement of cervical cancer treatment modalities, patient survival rates have improved significantly, particularly among younger patient populations. Therefore, the assessment of postoperative quality of life and sexual function is receiving increasing attention. Studies indicate that while C2-type/Type III radical hysterectomy enhances patient survival outcomes, it is frequently associated with a spectrum of postoperative physiological and psychological complications—including bladder dysfunction, intestinal dysmotility, lymphedema, peripheral neuropathy, and sexual dysfunction—which substantially compromise patients’ quality of life (QoL). Thus, when formulating treatment strategies, in addition to prioritizing oncological outcomes, postoperative complications, QoL, and sexual function should also be incorporated as critical considerations (6).

Studies have indicated that patients with a body mass index (BMI) greater than 25 kg/m² are more prone to postoperative complications (7). However, BMI fails to distinguish between muscle and fat compartments. Consequently, recent studies advocate for body composition indices that more accurately quantify the relative contributions of skeletal muscle and adipose tissue to total body weight (8, 9). Currently utilized methodologies for assessing body composition include, but are not limited to, CT, dual-energy X-ray absorptiometry (DXA), and bioelectrical impedance analysis (BIA). Cancer patients routinely undergo computed tomography (CT) scans for baseline staging and treatment response evaluation, while radiotherapy patients require CT simulation for treatment planning. These images provide an opportunity to extract body composition parameters. Consequently, CT-derived body composition measurements have been extensively investigated as significant prognostic indicators in cancer patients (10).

In the context of cancer, muscle loss constitutes a hallmark of disease-related malnutrition, arising from intertwined metabolic and inflammatory dysregulation rather than caloric deficit alone. Recent mechanistic insights indicate that this process involves a dual dysregulation of key pathways: the upregulation of catabolic systems (e.g., ubiquitin-proteasome and autophagy-lysosome via NF-κB) alongside the suppression of anabolic signaling (e.g., insulin/IGF-1 and mTOR). The resulting sarcopenia is a robust prognostic factor in cancer patients, significantly impacting treatment tolerance and survival outcomes (11). For patients with cervical cancer, the majority of studies indicate that sarcopenia or muscle loss during treatment correlates with adverse prognosis (12–17). Moreover, whether body composition influences postoperative complications in early-stage cervical cancer has not been clearly addressed. Evidence from endometrial and ovarian cancers suggested that altered body composition is associated with surgical complications, underscoring the need for further research in gynecologic malignancies (18).

Systemic inflammation also plays a pivotal role in cancer progression, influencing tumor initiation, invasion, and metastasis (19). Markers of systemic inflammation calculated from peripheral blood cell counts and serum albumin have been linked to poor surgical outcomes (20). The prognostic nutritional index (PNI), which integrates serum albumin and lymphocyte count, reflects both nutritional and inflammatory status. Previous studies across multiple cancer types have demonstrated that low PNI is associated with worse treatment response and poorer surgical outcomes (21–25).

Therefore, this study aimed to examine the relationship between CT-based body composition indices, systemic inflammatory markers, and postoperative complications in patients with early-stage cervical cancer.

## 2 Materials and methods

### 2.1 Patient selection

Patients diagnosed with cervical cancer who underwent surgical treatment at Gansu Provincial Maternity and Child Care Hospital or Gansu Provincial Central Hospital between July 2018 and December 2021 were retrospectively enrolled.

The inclusion criteria were as follows: (1) Pathologically confirmed squamous cell carcinoma, adenocarcinoma, or adenosquamous carcinoma; (2) FIGO 2018 stage IB1-IIA2; (3) Availability of complete clinical data, including preoperative abdominal CT scans with axial images at the level of the third lumbar vertebra (L3); (4) Underwent Querleu-Morrow type C1 or C2 radical hysterectomy; (5) Eastern Cooperative Oncology Group (ECOG) performance status of 0 or 1.

**Figure 1:**
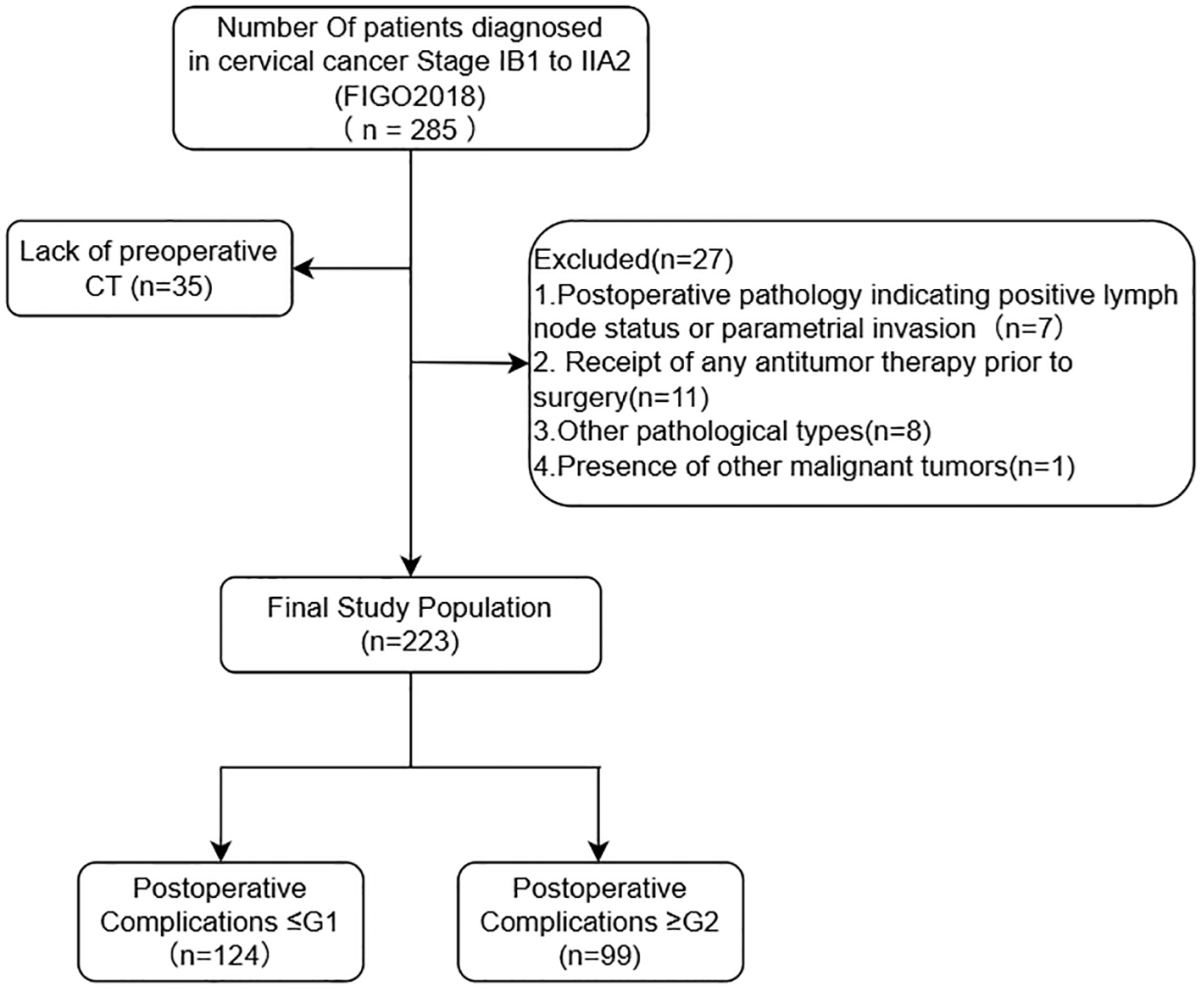
Flow diagram depicting selection of study population.

Exclusion criteria included: (1) Receipt of any antitumor therapy prior to surgery; (2) Postoperative pathology indicating positive lymph node status or parametrial invasion; (3) Presence of other malignant tumors; (4) Comorbidities such as hematological diseases, autoimmune disorders, acute or chronic infections, or other conditions likely to affect hematological parameters (**Figure 1**). The study protocol was approved by the Ethics Committee of Gansu Provincial Maternity and Child Care Hospital and Gansu Provincial Central Hospital.

### 2.2 Clinical information

All patients underwent computed tomography (CT) and hematological examinations within one week preoperatively (mean: 3.4 days and 4.1 days before surgery, respectively), with complete blood count and biochemical tests performed on the first postoperative day. Collected data included patient characteristics (age, height, weight), surgical complications, pathological characteristics (histological type, tumor size), postoperative adjuvant treatments, and hematological parameters (white blood cell count, neutrophil count, hemoglobin, platelet count, lymphocyte count, albumin). Axial CT images at the L3 level were analyzed using SliceOmatic 5.0 software to quantify muscle, visceral adipose tissue, subcutaneous adipose tissue, and intermuscular adipose tissue.

The selection of surgical approach was primarily determined by patient preferences and surgeon practice patterns, independent of body composition. Thirty-three patients who underwent laparoscopic surgery were enrolled in this study. Among them, one patient was diagnosed with stage IB2,one with stage IB3, and three with stage IIA1; these five patients all underwent surgery in 2018. The remaining patients were all classified as stage IB1. Subsequent to 2019, in alignment with the LACC trial and related studies, the multidisciplinary team (MDT) for cervical cancer at our institution established the following protocol: laparoscopic surgery was indicated for patients with stage IB1 disease or lower, whereas patients with higher stages underwent open surgery. All patients undergo routine lower extremity venous ultrasound within two weeks postoperatively to assess for the presence of postoperative thrombosis.

#### 2.2.1 Classification of surgical complications

Complications occurring within 3 months postoperatively were analyzed based on patients’ medical records and follow-up data. Postoperative complications were graded according to the Clavien-Dindo classification system. **Grade 1:** Any deviation from the normal postoperative course not requiring pharmacological, surgical, endoscopic, or radiological interventions (permitted therapies: antiemetics, antipyretics, analgesics, diuretics, electrolytes, and physiotherapy; also includes wound infections treated at bedside). **Grade 2:** Requires pharmacological treatment beyond Grade 1 complications, including blood transfusions or total parenteral nutrition. **Grade 3:** Requires surgical, endoscopic, or radiological intervention (3a: without general anesthesia; 3b: under general anesthesia). **Grade 4:** Life-threatening complication requiring intermediate care (IC) or management in an intensive care unit (ICU), including major central nervous system complications such as cerebral hemorrhage, ischemic stroke, and subarachnoid hemorrhage, but excluding transient ischemic attacks. **Grade 5:** Death (26).

### 2.3 Systemic inflammatory markers

Systemic inflammatory markers were calculated as follows, based on hematological parameters:

NLR (neutrophil-to-lymphocyte ratio) = neutrophil count ÷ lymphocyte count;
PLR (platelet-to-lymphocyte ratio) = platelet count ÷ lymphocyte count;
LMR (lymphocyte-to-monocyte ratio) = lymphocyte count ÷ monocyte count;
NPR (neutrophil-to-platelet ratio) = neutrophil count ÷ platelet count;
SII (systemic immune-inflammation index) = platelet count × NLR;
PNI (prognostic nutritional index) = albumin concentration (g/L) + 5 × lymphocyte count (×10^9^/L).

### 2.4 Body composition analysis

**Figure 2:**
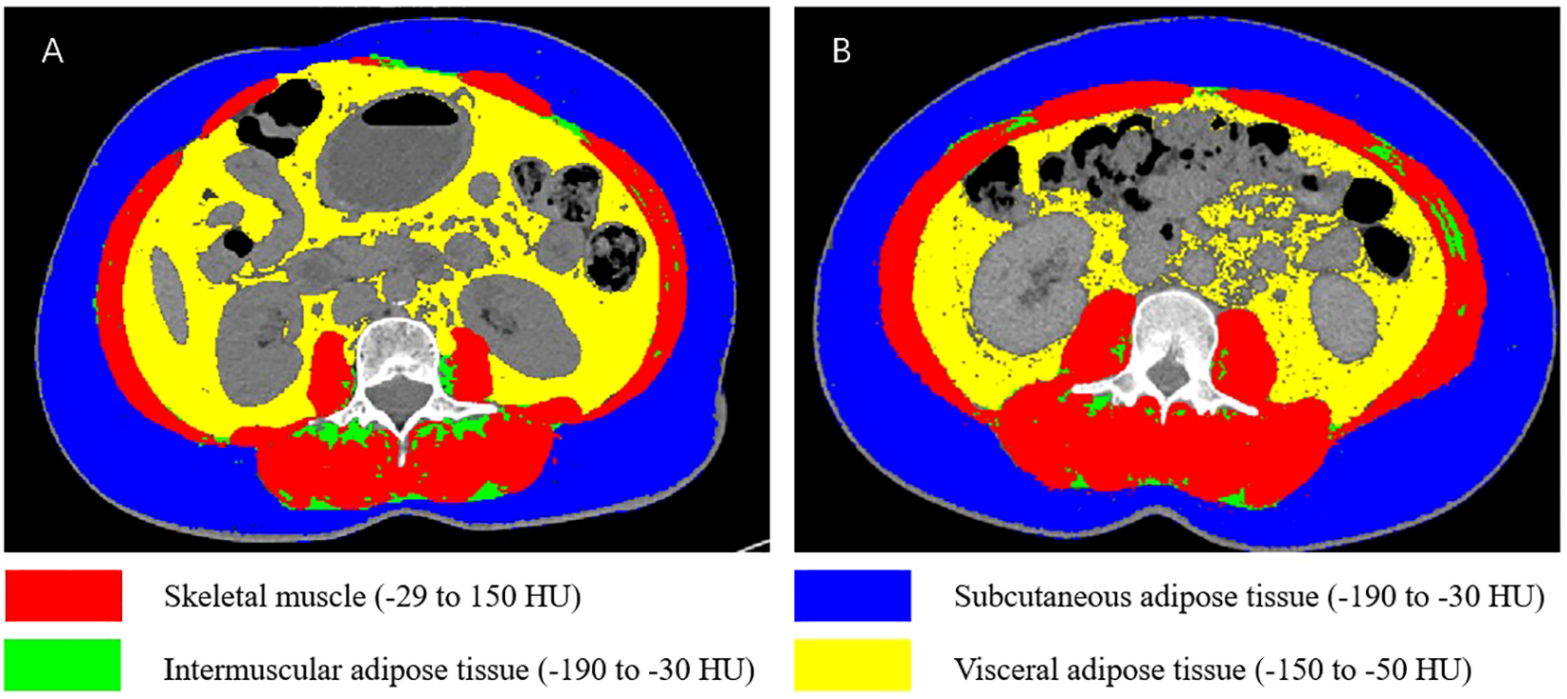
Schematic illustration of body composition at the L3 vertebral level. **(A)** Patient with sarcopenia and high VATI. **(B)** Patient without sarcopenia and low VATI.

CT-derived body composition compartments were identified using Hounsfield unit (HU) thresholds: skeletal muscle (-29 to 150 HU), subcutaneous adipose tissue (-190 to -30 HU), visceral adipose tissue (-150 to -50 HU), and intermuscular adipose tissue (-190 to -30 HU). The cross-sectional areas (cm²) of skeletal muscle, intermuscular adipose tissue, subcutaneous adipose tissue, and visceral adipose tissue were analyzed using SliceOmatic 5.0 software and normalized to height squared (m²) to calculate: skeletal muscle index (SMI), intermuscular adipose tissue index (IMATI), subcutaneous adipose tissue index (SATI), and visceral adipose tissue index (VATI) (**Figure 2**).

All CT images were segmented using SliceOmatic 5.0 software by a researcher with professional training and extensive experience in body composition analysis. The analyst was fully blinded to patients’ postoperative outcomes (complication severity) to ensure the objectivity of measurements. Among 50 randomly selected patients, the intra-observer coefficients of variation (CVs) for skeletal muscle area, intermuscular fat area, subcutaneous fat area, and visceral fat area were 1.2%, 1.4%, 1.3%, and 1.1%, respectively-consistent with findings from previous studies (12). These results indicate high reproducibility of body composition parameter measurements. Sarcopenia was defined using internationally widely adopted sex-specific cutoff values: for males, SMI< 43.0 cm²/m² (when body mass index [BMI] ≥ 25 kg/m²) or < 53.0 cm²/m² (when BMI < 25 kg/m²); for females, SMI < 41.0 cm²/m² (27). The study population of this paper consists of female patients. Sarcopenia was defined as a SMI< 41.0 cm²/m². To date, no optimal cut-off values have been established for IMATI, SATI, and VATI.

### 2.5 Statistical analysis

Normally distributed continuous variables were presented as mean ± standard deviation ( x ± s)and compared using the independent samples t-test. Non-normally distributed variables were expressed as median (interquartile range) [M (Q1, Q3)] and compared using the Mann-Whitney U test. Categorical variables were reported as numbers (expressed as percentages) and analyzed with the χ² test. Binary logistic regression was performed to examine the association between clinical factors and complications. Factors with a p-value < 0.1 were included in the multivariate regression analysis, followed by collinearity diagnosis with a variance inflation factor (VIF) threshold set at < 5. The Box-Tidwell test was used to assess the linearity assumption between continuous variables and logit(P), and the results were validated via Bonferroni correction. The Hosmer-Lemeshow test was performed to assess the calibration performance of the model, where a p-value > 0.05 was indicative of adequate calibration. The model’s discriminative ability was evaluated by computing the area under the curve (AUC) and its 95% confidence interval (95% CI). Additionally, the Nagelkerke R² was reported to quantify the model’s goodness of fit. To assess the robustness of the model, a sensitivity analysis adjusting for confounding factors was performed. Data analyses were conducted using IBM SPSS Statistics (version 27.0). A two-tailed p-value < 0.05 was considered statistically significant.

Given the exploratory retrospective study design of the present study, no *a priori* sample size calculation was performed. Instead, a *post hoc* power analysis was conducted to evaluate the study’s ability to detect the primary effect of interest. Given 99 complication events and an alpha level of 0.05 (two-tailed), the statistical power of this study to detect the associations between SMI (OR = 0.34), VATI (OR = 4.01, high vs. low), PNI (OR = 0.28, high vs. low) and complications was all > 90%. Additionally, the analysis demonstrated that, at 80% statistical power, the minimum detectable odds ratio (OR) for this study was approximately 1.8.

## 3 Results

1. A total of 223 patients were included, with a median age of 50 years. Specifically, 101 patients (45.3%) were classified as FIGO 2018 stage I, and 122 patients (54.7%) as stage II. Squamous cell carcinoma was the predominant pathological type (83%). Open surgery was performed in 190 patients (85.2%), and 162 (72.6%) received postoperative adjuvant therapy. Regarding nutritional status, only 4 patients (1.8%) were underweight, whereas 89 patients (39.9%) were overweight (**Table 1**).

**Table 1:** Patient characteristics.

Characteristics	N (%)
Age	50(29-70)
BMI	
18.5	4(1.8%)
18.5-23.9	130(58.3%)
24	89(39.9%)
FIGO stage	
IB1	65(29.1%)
IB2	22(9.9%)
IB3	14(6.3%)
IIA1	89(39.9%)
IIA2	33(14.8%)
Surgical method	
Laparotomy	190(85.2%)
Laparoscopy	33(14.8%)
Pathological type	
SCC	185(83%)
AC or ASC	38(17%)
Adjuvant treatment methods	
No	61(27.4%)
Radiotherapy ± chemotherapy	162(72.6%)

SCC, squamous cell carcinoma; AC, adenocarcinoma; ASC, adenosquamous carcinoma.

2. Postoperative Complications: Postoperative complications were mainly ≤Grade 1 (n = 124, 55.6%). Grade 2 complications occurred in 69 patients (30.9%), most commonly postoperative hemorrhage (n = 23, 10.3%), intestinal obstruction (n = 37, 16.6%), and infection (n = 54, 24.2%). Among 30 patients with Grade 3 complications, the most frequent event was secondary wound suture (n = 18, 8.1%). Additionally, 14 patients (6.3%) required surgical intervention for urinary tract obstruction or fistula, and one patient (0.4%) developed an enteric fistula. No Grade 4 or higher complications were observed (**Table 2**).

**Table 2:** Postoperative complications.

Postoperative complications type	Postoperative complications C-D grade		
	NC or G1	G2	≥G3
Postoperative hemorrhage	200	23	0
Wound-Related complications	199	5	18
Intestinal obstruction	186	37	0
Infection	171	54	0
Lymphocyst	217	6	0
Urological complications	209	0	14
VTE	29	0	0
Enteric fistula	222	0	1
Total	124	69	30

NC, No complications; VTE, Venous thromboembolism.

3. Patients were stratified into two groups (≤G1 vs. ≥G2) for comparative analysis. Compared with the ≤G1 group, patients with complications ≥G2 had significantly higher proportions of open surgery (93.9% vs. 75%), larger tumors (85.5% vs. 73.7%), greater intraoperative blood loss (300 [200, 600] vs. 200 [100, 400] mL), lower postoperative albumin levels (30.86 ± 4.15 g/L vs. 32.42 ± 3.31 g/L), and longer hospital stays (23 [19, 29] days vs. 17 [14, 20] days). All these differences were statistically significant (P < 0.05). In terms of body composition and nutritional status, the ≥G2 group had lower skeletal muscle index (SMI) (39.82 ± 5.33 vs. 41.26 ± 5.17 cm²/m²), higher visceral adipose tissue index (VATI) (32.29 [23.85, 56.71] vs. 28.33 [14.68, 43.70] cm²/m²), and lower prognostic nutritional index (PNI) (51.90 [49.60, 54.40] vs. 53.80 [50.36, 56.07]) compared with the ≤G1 group (P < 0.05). No significant differences were observed in age, BMI, FIGO stage, pathological type, or other systemic inflammatory markers between the two groups (**Table 3**).

**Table 3:** Comparison between patients with high-grade (≥grade II) and low-grade(≤gradeI) complications according to the Clavien-Dindo classification.

Characteristics	≤G1(n=124)	≥G2(n=99)	t/z/χ2	p
Age	49.72 ± 7.88	50.37 ± 9.38	-0.57	0.57
Surgical method			11.46	<0.01
Laparotomy	93(75%)	93(93.9%)		
Laparoscopy	27(25%)	6(6.1%)		
BMI			3.19	0.07
≤24	81(65.3%)	53(53.5%)		
>24	43(34.7%)	46(46.5%)		
FIGO stage			2.50	0.11
Stage I	62(50%)	39(39.4%)		
Stage II	62(50%)	60(60.6%)		
Tumor size			4.11	0.04
≤4cm	104(85.5%)	72(73.7%)		
>4cm	20(14.5%)	27(26.3%)		
Pathological type			0.16	0.69
SCC	104(83.9%)	81(81.8%)		
AC/ASC	20(16.1%)	18(18.2%)		
SMI	41.26 ± 5.17	39.82 ± 5.33	2.04	0.04
IMATI	4.39 ± 2.22	4.84 ± 2.34	-1.48	0.14
SATI	58.99 ± 21.86	62.40 ± 26.60	-1.05	0.30
VATI	28.33(14.68,43.70)	32.29(23.85,56.71)	-2.66	0.01
TP	72.35 ± 5.35	72.24 ± 5.98	0.15	0.89
Alb	45.62 ± 5.08	44.27 ± 3.54	2.24	0.03
HB	131.44 ± 16.37	127.12 ± 20.24	1.76	0.08
PLT	229(188,281)	205(172,278)	-1.70	0.09
NLR	2.35(1.65,3.12)	2.17(1.58,3.11)	-1.34	0.20
SII	97.19(62.73,148.94)	94.50(63.46,149.85)	-0.02	0.94
PLR	151.06(113.95,193.88)	145.30(109.80,183.18)	-1.09	0.28
LMR	4.75(3.40,6.53)	4.70(3.75,6.07)	-0.22	0.83
PNI	53.80(50.36,56.07)	51.90(49.60,54.40)	-2.73	0.01
Intraoperative blood loss	200(100,400)	300(200.600)	-2.99	<0.01
Postoperative Alb	32.42 ± 3.31	30.86 ± 4.15	3.12	<0.01
VTE			7.95	<0.01
Yes	10(8.1%)	21(21.2%)		
No	114(91.9%)	78(78.8%)		
Hospital stays	17(14.20)	23(19,29)	-7.12	<0.01

TP, total protein; Alb, albumin.

**Figure 3:**
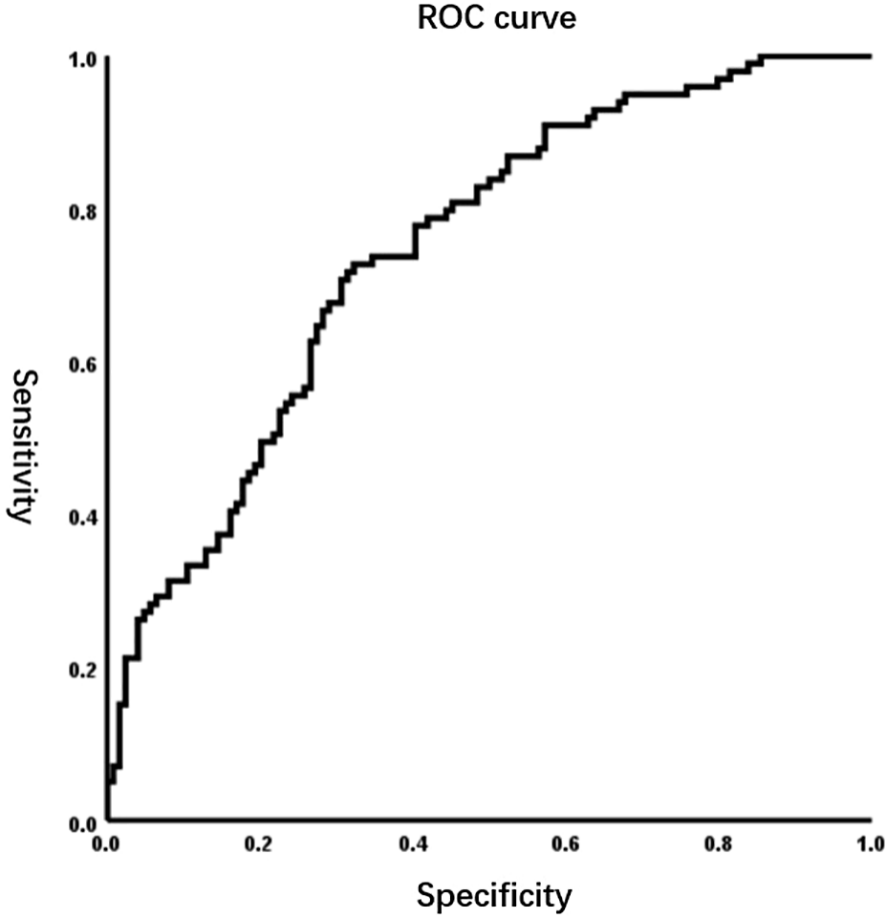
ROC curve for the prediction model, AUC = 0.745(95%CI 0.68-0.81).

4. Univariate logistic regression identified several factors, including tumor size > 4 cm, open surgery, greater intraoperative blood loss, low postoperative albumin, low SMI, high VATI, and low PNI, as risk factors for complications ≥G2. Factors with a p-value < 0.1 were included in the multivariable regression analysis. Collinearity diagnostics revealed that the variance inflation factor (VIF) ranged from 1.05 to 1.3. The Box-Tidwell test was performed, and Bonferroni correction (α = 0.05/9 = 0.0056) was applied to verify the linearity assumption between all continuous variables and logit(P). Results confirmed that the linear relationship held for all variables (all p > 0.0056). The Enter method was employed as the modeling strategy, where all candidate variables were included in the regression model simultaneously. Multivariate analysis confirmed that low SMI (OR = 0.34, 95% CI: 0.18–0.64, P < 0.001), high VATI (OR = 1.04, 95% CI: 1.02–1.06, P < 0.001), and low PNI (OR = 0.91, 95% CI: 0.84–0.98, P = 0.008) were independent predictors of higher complication grades (**Table 4**). The Hosmer-Lemeshow test indicated good model fit (χ² = 6.85, P = 0.55). The model demonstrated discriminative ability with an area under the receiver operating characteristic (ROC) curve (AUC) of 0.745 (95% CI: 0.68-0.81) (**Figure 3**). The Nagelkerke R² value was 0.245.

**Table 4:** Univariate and multivariate logistic regression analyses of risk factors for ≥grade 2 postoperative complications.

Variables	≥Grade 2 postoperative complications					
	Univariable			Multivariable		
	OR	95%CI	P	OR	95%CI	P
Age (Per 1 year)	1.01	0.98-1.04	0.569			
BMI (Per 1 kg/m^2^)	1.04	0.95-1.14	0.383			
FIGO Stage (Stage II vs Stage I)	1.54	0.90-2.63	0.115			
Tumor size(>4cm vs ≤ 4cm)	1.95	1.02-3.74	0.045	1.57	0.75-3.25	0.229
Pathological type (AC/ASC vs SCC)	1.09	0.55-2.18	0.808			
Surgical method (Laparoscopy vs Laparotomy)	0.36	0.16-0.81	0.013	0.49	0.20-1.22	0.124
Intraoperative blood loss (Per 100 mL)	1.10	1.03-1.18	0.009	1.05	0.96-1.15	0.312
Postoperative Alb (Per 1 g/L)	0.89	0.83-0.96	0.003	0.94	0.86-1.03	0.217
SMI (Non-sarcopenia vs sarcopenia)	0.49	0.29-0.85	0.012	0.34	0.18-0.64	0.001
IMATI (Per 1 cm2/m2)	1.09	0.97-1.23	0.143			
SATI (Per 1 cm2/m2)	1.01	0.99-1.02	0.295			
VATI (Per 1 cm2/m2)	1.02	1.01-1.04	0.004	1.04	1.02-1.06	0.001
NLR	0.88	0.73-1.07	0.198			
SII	1.00	1.00-1.01	0.528			
PLR	1.00	0.99-1.01	0.434			
LMR	1.00	0.88-1.14	0.991			
PNI	0.93	0.97-0.99	0.018	0.91	0.84-0.98	0.008
Interaction term analysis						
SMI × surgical method				0.89	0.72-1.10	0.27
VATI × surgical method				1.02	0.95-1.09	0.64

Given the potential interaction effects of Surgical methods, we performed a corresponding interaction analysis to account for such confounding. In the multivariate regression model incorporating interaction terms (SMI × surgical method, VATI × surgical method), neither the SMI × surgical method interaction term (OR = 0.89, 95% CI: 0.72–1.10, p = 0.27) nor the VATI × surgical method interaction term (OR = 1.02, 95% CI: 0.95–1.09, p = 0.64) achieved statistical significance. Notably, despite variable centering, the statistical power of this conclusion may be constrained by inherent multicollinearity in the interaction term model (VIF > 10). To complement the interaction term analysis, we performed an exploratory stratified analysis (see **Supplementary Table S1**). This stratified analysis revealed consistent effect directions of SMI and VATI across the two subgroups. However, the laparoscopic subgroup (n = 33) had a relatively small sample size, leading to imprecise effect estimates that did not achieve statistical significance. This observation corroborates the non-significant findings from the interaction term analysis.

5. Sensitivity analysis (**Table 5**).

**Table 5:** Summary of sensitivity analyses for key findings.

Analysis dimension	Detailed analysis	SMI effect (OR,95%CI)	VATI effect (OR,95% CI)	PNI effect (OR,95% CI)
SMI	As a Continuous Variable (Per 1 cm2/m2)	0.90(0.85-0.96)		
	Per 5 cm2/m2	0.61(0.45-0.82)		
VATI	Per5 cm2/m2		1.22(1.10-1.34)	
	Tertile (cm2/m2)			
	≤23.64		Ref	
	23.64-39.71		3.78(1.74-8.20)	
	≥39.71		4.01(1.77-9.07)	
PNI	Per 5 cm2/m2			0.61(0.42-0.88)
	Tertile(cm2/m2)			
	≤51.03			Ref
	51.03-54.50			0.90(0.44-1.83)
	≥54.50			0.28(0.13-0.64)
Confounding Control	Adjusted for Blood Loss and Tumor Size	0.35(0.19-0.66)	1.04(1.02-1.06)	0.91(0.85-0.98)
	Adjusted for Postoperative Blood Transfusion	0.37(0.19-0.73)	1.04(1.02-1.06)	0.91(0.84-0.99)

Alternative Modeling of SMI: When analyzed as a continuous variable, SMI retained a significant protective effect (per 1-unit increase: OR = 0.90, 95% CI: 0.85–0.96; per 5-unit increase: OR = 0.61, 95% CI: 0.45–0.82).

Robustness Testing for VATI and PNI: After converting VATI to tertiles, a clear dose-response relationship was identified (high vs. low tertile: OR = 4.01, 95% CI: 1.77–9.07). Similarly, tertile conversion of PNI revealed a protective effect (high vs. low tertile: OR = 0.28, 95% CI: 0.13–0.64).

Adjustment for key confounding factors: After additional adjustment for intraoperative blood loss, tumor size, and postoperative blood transfusion, the effect estimates for SMI, VATI, and PNI exhibited only minimal changes and remained statistically significant, indicating robustness of the associations after accounting for potential clinical confounders.

## 4 Discussion

Previous studies on surgical complications in cervical cancer patients have primarily focused on clinicopathological factors and surgical approaches. However, the potential impact of body composition on postoperative complications in early-stage cervical cancer has not been previously reported. Our study demonstrates that sarcopenia, high VATI, and low PNI are significantly associated with an increased complication risk. These findings suggest that patients with favorable body composition profiles experienced fewer postoperative complications, highlighting the potential clinical benefits of interventions aimed at improving body composition.

Surgical approaches for early-stage cervical cancer include open, laparoscopic, and robotic procedures. The LACC trial reported higher recurrence and mortality rates following minimally invasive surgery (laparoscopic and robotic) compared with open surgery, thereby establishing open surgery as the standard of care for early-stage disease (28, 29). Accordingly, the majority of patients in our cohort underwent open surgery (83.4%). Other studies have demonstrated comparable postoperative complication rates between laparoscopic and open surgery (30, 31). However, given its advantages in reducing intraoperative blood loss and shortening hospital stay, laparoscopic may be considered for selective use in low-risk patients—defined as those with tumor size <2 cm, absence of lymphovascular invasion, and depth of invasion <10 mm—provided the oncological safety principles are strictly adhered to (32). This study found no significant effect modification by surgical approach on the association between body composition and postoperative complications. The consistent direction of effects observed in both interaction and stratified analyses suggests that ameliorating sarcopenia and reducing visceral adiposity may confer clinical benefits across all surgical patients, regardless of procedure type. Larger-scale prospective studies are warranted to precisely estimate the effect size of this association specifically among patients undergoing laparoscopic surgery. To precisely evaluate the independent effect of body composition on postoperative complications, this study excluded patients with positive lymph nodes or parametrial involvement based on postoperative pathological findings. This methodological decision was primarily based on the following rationale: advanced lesions typically necessitate more extensive and radical lymph node dissection or parametrial tissue resection intraoperatively, and these expanded surgical procedures are established independent risk factors for postoperative complications. Including these patients could increase the incidence of severe complications. This exclusion criterion may introduce selection bias, thereby limiting the generalizability of the study findings to some extent. Future studies should aim to validate the predictive value of body composition indicators across different FIGO stages in cohorts that include more advanced disease cases.

The PNI, first proposed by Buzby et al. (33), integrates serum albumin levels and lymphocyte count, thereby reflecting both nutritional status and systemic inflammation. Due to its simplicity and clinical utility, PNI has been widely applied in various cancers. Substantial evidence indicates that low PNI is an independent predictor of short-term postoperative complications in malignancies such as colorectal (23), gastric (24), and hepatocellular carcinoma (25). However, studies on the role of PNI in gynecological cancers remain limited. For example, a large cohort of 1,000 gynecological cancer patients (11.4% cervical, 55.1% ovarian, and 33.5% endometrial) demonstrated that PNI > 40 was significantly associated with lower complication rates (21). Similarly, in advanced ovarian cancer, PNI < 45 correlated with increased 90-day mortality and a higher incidence of severe complications (34). In the context of early-stage cervical cancer, our findings confirm that low PNI is an independent risk factor for postoperative complications, underscoring its potential value in surgical risk stratification for gynecological malignancies.

Skeletal muscle serves not only as a protein reservoir but also as an important site for immune regulation. Myokines, secreted by muscle cells, modulate immune responses, and sarcopenia is frequently accompanied by a chronic low-grade inflammatory state. Emerging evidence indicates that high-risk HPV infection alone can trigger both local and systemic inflammatory responses and oxidative stress. The “chronic inflammatory baseline” induced by HPV may act synergistically with the malnutrition and systemic inflammation observed in this study, collectively amplifying the physiological stress response to surgical trauma and thereby increasing the risk of postoperative complications such as infection and impaired anastomotic healing (35). These factors likely underpin the heightened risk of postoperative complications in patients with sarcopenia, serving as a key pathophysiological basis (11). However, the relationship between sarcopenia and complications in gynecological cancers remains controversial. For instance, studies defining sarcopenia by psoas muscle area reported longer hospital stays but no significant differences in surgical complications among patients with endometrial cancer (36) and higher in-hospital mortality and non-surgical complications in ovarian cancer, without significant differences in surgery-related complications (37). By contrast, another study of 250 endometrial cancer patients used CT-derived skeletal muscle index and found that both skeletal muscle mass and high-radiodensity skeletal muscle index were associated with greater complications (38). Similarly, bioelectrical impedance analysis in ovarian and endometrial cancer suggested that muscle mass < 21.8 kg and skeletal muscle index (SMI) < 27% were linked to severe complications in univariate, but not multivariate, analysis (39). These inconsistent findings, compared with the more robust associations observed in gastrointestinal cancers, may reflect differences in body composition measurement techniques and diagnostic criteria for sarcopenia. Recently, artificial intelligence-based whole-body composition analysis has gained traction, offering greater efficiency and consistency in body composition assessment, and may help establish standardized methods for future research (40).

VATI also significantly increases intraoperative challenges. Excess visceral fat restricts surgical field exposure, obscures visualization of critical anatomical structures, and limits instrument mobility and precision during deep procedures such as lymph node dissection. These factors elevate the risk of accidental injury to adjacent organs and perioperative complications. In addition, visceral fat is a metabolically active tissue. The chronic low-grade inflammation and insulin resistance caused by its accumulation can damage the physiological reserves and stress response capacity of patients. This, in conjunction with the mechanical challenges of surgery, jointly increases the risk of postoperative complications (41). Previous studies support these observations: in endometrial cancer, increased visceral fat was independently predicted to convert to open surgery (42). Alejandro et al. demonstrated that patients with sagittal abdominal diameter (SAD) > 246 mm, as measured by MRI, had markedly higher complication rates (69% vs. 31%) (43). Similarly, visceral obesity has been associated with higher complication rates in advanced ovarian cancer undergoing cytoreductive surgery (44). In line with these findings, our results suggest that proactive surgical strategies are warranted for patients with high VATI. Such measures include using longer instruments for deep surgery, clearly identifying anatomical landmarks obscured by adipose tissue, carefully planning surgical approaches and lymphadenectomy pathways, and thoroughly discussing the possibility of conversion to open surgery with patients. These tailored approaches may be crucial for reducing complication risk in this population.

The predictive model developed in this study exhibited acceptable discriminative ability (AUC = 0.745) and favorable calibration, while accounting for a substantial proportion of outcome variance (Nagelkerke R² = 0.245). This finding suggests that integrating objective metrics of body composition (SMI, VATI) and systemic inflammatory markers (PNI) substantially enhances the assessment of postoperative complication risk, relative to evaluations based solely on clinical experience. Notably, a series of rigorous sensitivity analyses—including outlier exclusion, adoption of alternative variable modeling frameworks (continuous vs. categorical), and additional adjustment for key confounding variables such as intraoperative blood loss—validated the robustness of the core findings: the predictive effects of SMI and VATI on complications remained stable and consistent across diverse analytical contexts. The robustness of these findings, coupled with validation of the linearity assumption, collectively strengthens the statistical reliability of the study’s conclusions. In summary, these findings not only demonstrate that body composition is a robust predictor independent of surgery-related factors, but also suggest that it may represent an actionable target for preoperative optimization aimed at mitigating surgical risk. Future studies should aim to validate these metrics in prospective cohorts, integrate them into clinical workflows, and further explore intervention strategies to optimize surgical outcomes by targeting improvements in patients’ body composition.

A key strength of our study is that it is the first to integrate body composition and systemic inflammatory markers to investigate complications in patients with early-stage cervical cancer. Additionally, we performed a comprehensive suite of sensitivity analyses and rigorous model diagnostics. Results indicate that our core findings remained robust across varying model specifications, different subgroups, and following the exclusion of key confounding factors; further, the model exhibited adequate statistical power. This study has several limitations. First, as a single-center retrospective analysis, it is susceptible to information bias. Second, the sample size of the laparoscopic surgery subgroup is relatively small, which may have limited statistical power in stratified analyses and potentially reduced the generalizability of the findings. Third, while multiple confounding factors were adjusted for, the possibility of residual confounding cannot be entirely ruled out.

## 5 Conclusion

In summary, the findings of this study demonstrate that sarcopenia, a high visceral adipose tissue index (VATI), and a low prognostic nutritional index (PNI) are independently associated with an increased risk of postoperative complications in patients with early-stage cervical cancer. Systematic preoperative assessment of these metrics can facilitate the identification of high-risk patients, enable the development of individualized surgical protocols and nutritional intervention strategies, and may ultimately mitigate the risk of postoperative complications.

## Data Availability

The datasets presented in this article are not readily available because The data in this article must be approved by the institution before it can be provided. Requests to access the datasets should be directed to Lipeng Ding, 1396613501@qq.com.

## References

[1] Sung H Ferlay J Siegel RL Laversanne M Soerjomataram I Jemal A . Global cancer statistics 2020: GLOBOCAN estimates of incidence and mortality worldwide for 36 cancers in 185 countries. CA Cancer J Clin. (2021) 71:209–49. doi: 10.3322/caac.21660, PMID: 33538338

[2] Singh D Vignat J Lorenzoni V Eslahi M Ginsburg O Lauby-Secretan B . Global estimates of incidence and mortality of cervical cancer in 2020: a baseline analysis of the WHO Global Cervical Cancer Elimination Initiative. Lancet Glob Health. (2023) 11:e197–206. doi: 10.1016/S2214-109X(22)00501-0, PMID: 36528031 PMC9848409

[3] Lin S Gao K Gu S You L Qian S Tang M . Worldwide trends in cervical cancer incidence and mortality, with predictions for the next 15 years. Cancer. (2021) 127:4030–9. doi: 10.1002/cncr.33795, PMID: 34368955

[4] Abu-Rustum NR Yashar CM Arend R Barber E Bradley K Brooks R . NCCN guidelines^®^ Insights: cervical cancer, version 1.2024. J Natl Compr Canc Netw. (2023) 21:1224–33. doi: 10.6004/jnccn.2023.0062, PMID: 38081139

[5] Vázquez-Vicente D Boria F Castellanos T Gutierrez M Chacon E Manzour N . SUCCOR morbidity: complications in minimally invasive versus open radical hysterectomy in early cervical cancer. Int J Gynecol Cancer. (2024) 34:203–8. doi: 10.1136/ijgc-2023-004657, PMID: 38669163

[6] Plotti F Sansone M Di Donato V Antonelli E Altavilla T Angioli R . Quality of life and sexual function after type C2/type III radical hysterectomy for locally advanced cervical cancer: a prospective study. J Sex Med. (2011) 8:894–904. doi: 10.1111/j.1743-6109.2010.02133.x, PMID: 21143414

[7] Ersoy E Evliyaoğlu Ö Erol O Ersoy AÖ Akgül MA Haberal A . Effects of the morbid obesity and skin incision choices on surgical outcomes in patients undergoing total abdominal hysterectomy. Turk J Obstet Gynecol. (2016) 13:189–95. doi: 10.4274/tjod.67864, PMID: 28913120 PMC5558291

[8] Cakir H Heus C Verduin WM Lak A Doodeman HJ Bemelman WA . Visceral obesity, body mass index and risk of complications after colon cancer resection: A retrospective cohort study. Surgery. (2015) 157:909–15. doi: 10.1016/j.surg.2014.12.012, PMID: 25708142

[9] Mazzoccoli G . Body composition: Where and when. Eur J Radiol. (2016) 85:1456–60. doi: 10.1016/j.ejrad.2015.10.020, PMID: 26564096

[10] Bates DDB Pickhardt PJ . CT-derived body composition assessment as a prognostic tool in oncologic patients: from opportunistic research to artificial intelligence-based clinical implementation. AJR Am J Roentgenol. (2022) 219:671–80. doi: 10.2214/AJR.22.27749, PMID: 35642760

[11] Liu D Wang S Liu S Wang Q Che X Wu G . Frontiers in sarcopenia: Advancements in diagnostics, molecular mechanisms, and therapeutic strategies. Mol Aspects Med. (2024) 97:101270. doi: 10.1016/j.mam.2024.101270, PMID: 38583268

[12] Lee J Chang CL Lin JB Wu MH Sun FJ Jan YT . Skeletal muscle loss is an imaging biomarker of outcome after definitive chemoradiotherapy for locally advanced cervical cancer. Clin Cancer Res. (2018) 24:5028–36. doi: 10.1158/1078-0432.CCR-18-0788, PMID: 29959140

[13] Aichi M Hasegawa S Kurita Y . Low skeletal muscle mass predicts poor prognosis for patients with stage III cervical cancer on concurrent chemoradiotherapy. Nutrition. (2023) 109:111966. doi: 10.1016/j.nut.2022.111966, PMID: 36731243

[14] Kiyotoki T Nakamura K Haraga J Omichi C Ida N Saijo M . Sarcopenia is an important prognostic factor in patients with cervical cancer undergoing concurrent chemoradiotherapy. Int J Gynecol Cancer. (2018) 28:168–75. doi: 10.1097/IGC.0000000000001127, PMID: 29040185

[15] Matsuoka H Nakamura K Matsubara Y Ida N Nishida T Ogawa C . Sarcopenia is not a prognostic factor of outcome in patients with cervical cancer undergoing concurrent chemoradiotherapy or radiotherapy. Anticancer Res. (2019) 39:933–9. doi: 10.21873/anticanres.13196, PMID: 30711978

[16] Han Q Kim SI Yoon SH Kim TM Kang HC Kim HJ . Impact of computed tomography-based, artificial intelligence-driven volumetric sarcopenia on survival outcomes in early cervical cancer. Front Oncol. (2021) 11:741071. doi: 10.3389/fonc.2021.741071, PMID: 34631578 PMC8499694

[17] Lee J Lin JB Chen TC Jan YT Sun FJ Chen YJ . Progressive skeletal muscle loss after surgery and adjuvant radiotherapy impact survival outcomes in patients with early stage cervical cancer. Front Nutr. (2022) 8:773506. doi: 10.3389/fnut.2021.773506, PMID: 35127782 PMC8810512

[18] Heus C Stelten S Kenter GG Buffart LM van Lonkhuijzen LRCW . Body composition and peri- and postoperative complications in patients with gynaecological Malignancies: A systematic review. Gynecol Oncol. (2024) 190:131–8. doi: 10.1016/j.ygyno.2024.08.014, PMID: 39182424

[19] Elinav E Nowarski R Thaiss CA Hu B Jin C Flavell RA . Inflammation-induced cancer: crosstalk between tumours, immune cells and microorganisms. Nat Rev Cancer. (2013) 13:759–71. doi: 10.1038/nrc3611, PMID: 24154716

[20] Košec A Solter D Ribić A Knežević M Vagić D Pegan A . Systemic inflammatory markers as predictors of postoperative complications and survival in patients with advanced head and neck squamous cell carcinoma undergoing free-flap reconstruction. J Oral Maxillofac Surg. (2022) 80:744–55. doi: 10.1016/j.joms.2021.12.011, PMID: 35032441

[21] Bermúdez-Pineda B García-Luna MÁ Oñate-Ocaña LF Morales-Piélago GF Cantú-De León DF Reynoso-Noverón N . Prognostic nutritional index as a predictor of surgical complications in women with gynecological cancer. Int J Gynecol Cancer. (2025) 35:101860. doi: 10.1136/ijgc-2024-005873, PMID: 39366718

[22] Wang HB Xu XT Tian MX Ding CC Tang J Qian Y . Prognostic values of the prognostic nutritional index, geriatric nutritional risk index, and systemic inflammatory indexes in patients with stage IIB-III cervical cancer receiving radiotherapy. Front Nutr. (2023) 10:1000326. doi: 10.3389/fnut.2023.1000326, PMID: 36937347 PMC10017984

[23] Sun G Li Y Peng Y Lu D Zhang F Cui X . Impact of the preoperative prognostic nutritional index on postoperative and survival outcomes in colorectal cancer patients who underwent primary tumor resection: a systematic review and meta-analysis. Int J Colorectal Dis. (2019) 34:681–9. doi: 10.1007/s00384-019-03241-1, PMID: 30680451

[24] Jing Y Ren M Li X Sun X Xiao Y Xue J . The effect of systemic immune-inflammatory index (SII) and prognostic nutritional index (PNI) in early gastric cancer. J Inflammation Res. (2024) 17:10273–87. doi: 10.2147/JIR.S499094, PMID: 39654858 PMC11625636

[25] Zhang H Li D Li J . Prognostic significance of preoperative prognostic nutritional index in hepatocellular carcinoma after curative hepatectomy: a meta-analysis and systemic review. Front Nutr. (2024) 11:1433528. doi: 10.3389/fnut.2024.1433528, PMID: 39764415 PMC11700793

[26] Clavien PA Barkun J de Oliveira ML Vauthey JN Dindo D Schulick RD . The Clavien-Dindo classification of surgical complications: five-year experience. Ann Surg. (2009) 250:187–96. doi: 10.1097/SLA.0b013e3181b13ca2, PMID: 19638912

[27] Martin L Birdsell L Macdonald N Reiman T Clandinin MT McCargar LJ . Cancer cachexia in the age of obesity: skeletal muscle depletion is a powerful prognostic factor, independent of body mass index. J Clin Oncol. (2013) 31:1539–47. doi: 10.1200/JCO.2012.45.2722, PMID: 23530101

[28] Ramirez PT Frumovitz M Pareja R Lopez A Vieira M Ribeiro R . Minimally invasive versus abdominal radical hysterectomy for cervical cancer. N Engl J Med. (2018) 379:1895–904. doi: 10.1056/NEJMoa1806395, PMID: 30380365

[29] Ramirez PT Robledo KP Frumovitz M Pareja R Ribeiro R Lopez A . LACC trial: final analysis on overall survival comparing open versus minimally invasive radical hysterectomy for early-stage cervical cancer. J Clin Oncol. (2024) 42:2741–6. doi: 10.1200/JCO.23.02335, PMID: 38810208

[30] Obermair A Asher R Pareja R Frumovitz M Lopez A Moretti-Marques R . Incidence of adverse events in minimally invasive vs open radical hysterectomy in early cervical cancer: results of a randomized controlled trial [published correction appears. Am J Obstet Gynecol. (2020) 222:249.e1–249.e10. doi: 10.1016/j.ajog.2019.09.036, PMID: 31586602 PMC7181470

[31] Levin G Ramirez PT Wright JD Slomovitz BM Hamilton KM Schneyer RJ . Approach to radical hysterectomy for cervical cancer after the Laparoscopic Approach to Cervical Cancer trial and associated complications: a National Surgical Quality Improvement Program study. Am J Obstet Gynecol. (2025) 232:208.e1–208.e11. doi: 10.1016/j.ajog.2024.08.008, PMID: 39151769

[32] Di Donato V Bogani G Casarin J Ghezzi F Malzoni M Falcone F . Ten-year outcomes following laparoscopic and open abdominal radical hysterectomy for “low-risk” early-stage cervical cancer: A propensity-score based analysis. Gynecol Oncol. (2023) 174:49–54. doi: 10.1016/j.ygyno.2023.04.030, PMID: 37149905

[33] Buzby GP Mullen JL Matthews DC Hobbs CL Rosato EF . Prognostic nutritional index in gastrointestinal surgery. Am J Surg. (1980) 139:160–7. doi: 10.1016/0002-9610(80)90246-9, PMID: 7350839

[34] Fumagalli D Sonik R De Vitis LA Rossi V Bazzurini L McGree ME . Evaluating nutrition in advanced ovarian cancer: which biomarker works best? Gynecol Oncol. (2024) 188:97–102. doi: 10.1016/j.ygyno.2024.06.021, PMID: 38943693

[35] Terrinoni M Golia D’Augè T Mascellino G Adinolfi F Palisciano M Rossetti D . Human papillomavirus across the reproductive lifespan: an integrative review of fertility, pregnancy outcomes, and fertility-sparing management. Med (Kaunas). (2025) 61:1499. doi: 10.3390/medicina61081499, PMID: 40870544 PMC12388228

[36] Bilir F Özgül E Elaziz B Arıöz DT . Clinical implication of preoperative psoas muscle area in endometrial cancer patients. Rev Assoc Med Bras (1992). (2021) 67:1759–63. doi: 10.1590/1806-9282.20210364, PMID: 34909946

[37] Can E Sönmez S Konal M Şirinoglu HA Seyhan NA Akbayır Ö . Does sarcopenia predict perioperative mortality in patients with advanced ovarian cancer? Eur Rev Med Pharmacol Sci. (2022) 26:9409–15. doi: 10.26355/eurrev_202212_30692, PMID: 36591849

[38] Silva de Paula N de Aguiar Bruno K Azevedo Aredes M Villaça Chaves G . Sarcopenia and skeletal muscle quality as predictors of postoperative complication and early mortality in gynecologic cancer. Int J Gynecol Cancer. (2018) 28:412–20. doi: 10.1097/IGC.0000000000001157, PMID: 29266018

[39] Sehouli J Mueller K Richter R Anker M Woopen H Rasch J . Effects of sarcopenia and malnutrition on morbidity and mortality in gynecologic cancer surgery: results of a prospective study. J Cachexia Sarcopenia Muscle. (2021) 12:393–402. doi: 10.1002/jcsm.12676, PMID: 33543597 PMC8061344

[40] Raia G Del Grande M Colombo I Nerone M Manganaro L Gasparri ML . Whole-body composition features by computed tomography in ovarian cancer: pilot data on survival correlations. Cancers (Basel). (2023) 15:2602. doi: 10.3390/cancers15092602, PMID: 37174067 PMC10177066

[41] Ibrahim MM . Subcutaneous and visceral adipose tissue: structural and functional differences. Obes Rev. (2010) 11:11–8. doi: 10.1111/j.1467-789X.2009.00623.x, PMID: 19656312

[42] Palomba S Zupi E Russo T Oppedisano R Manguso F Falbo A . Presurgical assessment of intraabdominal visceral fat in obese patients with early-stage endometrial cancer treated with laparoscopic approach: relationships with early laparotomic conversions. J Minim Invasive Gynecol. (2007) 14:195–201. doi: 10.1016/j.jmig.2006.09.019, PMID: 17368256

[43] Correa-Paris A Gorraiz Ochoa V Hernandez Gutiérrez A Gilabert Estellés J Díaz-Feijoo B Gil-Moreno A . Simple radiologic assessment of visceral obesity and prediction of surgical morbidity in endometrial cancer patients undergoing laparoscopic aortic lymphadenectomy: A reliability and accuracy study. J Obstet Gynaecol Res. (2023) 49:988–97. doi: 10.1111/jog.15528, PMID: 36593218

[44] Heus C Smorenburg A Stoker J Rutten MJ Amant FCH van Lonkhuijzen LRCW . Visceral obesity and muscle mass determined by CT scan and surgical outcome in patients with advanced ovarian cancer. A retrospective cohort study. Gynecol Oncol. (2021) 160:187–92. doi: 10.1016/j.ygyno.2020.10.015, PMID: 33393479

