# Supplementary material for: The impact of body composition and systemic inflammatory markers on postoperative complications in early-stage cervical cancer
Source: Front Oncol. 2026 Jan 6;15:1696383. doi: 10.3389/fonc.2025.1696383 (PMC12815836; doi:10.3389/fonc.2025.1696383)
Supplement: Supplementary file 1 [file DataSheet1.pdf]

STROBE Statement—checklist of items that should be included in reports of observational studies

	Item No.	Recommendation	Page No.	Relevant text from manuscript
**Title and abstract**	1	(*a*) Indicate the study's design with a commonly used term in the title or the abstract	Page 1, Paragraph 2	Methods: This study is a retrospective cohort study based on early-stage cervical cancer.
		(*b*) Provide in the abstract an informative and balanced summary of what was done and what was found	Page 1 Paragraph 1-4	Abstract (Objective/ Methods/ Results/ Conclusion)
**Introduction**				
Background/rationale	2	Explain the scientific background and rationale for the investigation being reported	Page 2 Paragraph 2-5	Relevant paragraphs in the Introduction section
Objectives	3	State specific objectives, including any prespecified hypotheses	Page 3 Paragraph 1	Therefore, this study aimed to examine the relationship between CT-based body composition indices, systemic inflammatory markers, and postoperative complications in patients with early-stage cervical cancer.
**Methods**				
Study design	4	Present key elements of study design early in the paper	Page 1, Paragraph 2	This study is a retrospective cohort study based on early-stage cervical cancer. A retrospective analysis was conducted on 223 patients with early-stage cervical cancer treated at our center between July 2018 and December 2021.
Setting	5	Describe the setting, locations, and relevant dates, including periods of recruitment, exposure, follow-up, and data collection	Page 3, Paragraph 2-4	Materials and Methods Patient Selection section

Participants	6	(*a*) *Cohort study*—Give the eligibility criteria, and the sources and methods of selection of participants. Describe methods of follow-up<br>*Case-control study*—Give the eligibility criteria, and the sources and methods of case ascertainment and control selection. Give the rationale for the choice of cases and controls<br>*Cross-sectional study*—Give the eligibility criteria, and the sources and methods of selection of participants	Page 3, Paragraph 4-7	Materials and Methods Patient Selection section and Clinical Information Selection section
		(*b*) *Cohort study*—For matched studies, give matching criteria and number of exposed and unexposed<br>*Case-control study*—For matched studies, give matching criteria and the number of controls per case	NA	This was not a matched cohort study.
Variables	7	Clearly define all outcomes, exposures, predictors, potential confounders, and effect modifiers. Give diagnostic criteria, if applicable	Page 3-4	Materials and Methods Classification of Surgical Complications/ Systemic Inflammatory Markers/ Body Composition Analysis section
Data sources/ measurement	8*	For each variable of interest, give sources of data and details of methods of assessment (measurement). Describe comparability of assessment methods if there is more than one group	Page 3-4	Materials and Methods Systemic Inflammatory Markers/ Body Composition Analysis section
Bias	9	Describe any efforts to address potential sources of bias	Page 4, Paragraph 4; Page 4, Paragraph 4; Page 5, Paragraph 1; Page 6, Paragraph 3; Page 9, Paragraph 2;	The CT analyst was blinded to patient outcomes. *(Methods, 'Body Composition Analysis' section)*<br>Intra-observer reproducibility was assessed in a random sample of 50 scans, demonstrating a CV of <1.5% for all tissue areas. *(Methods, 'Body Composition Analysis' section)*<br>Multivariable logistic regression

				was used to adjust for age, BMI, ASA score, tumor stage, and surgical approach. *(Methods, 'Statistical Analysis' section)*<br>A sensitivity analysis excluding patients who received postoperative blood transfusions (n=22) was performed, and the associations for SMI, VATI, and PNI remained significant."*(Results, 'Sensitivity Analysis' section)*<br>The primary limitation of this study is its retrospective nature, which precludes causal inference and may introduce unmeasured confounding. However, the use of objective CT measurements and comprehensive adjustment for known clinical factors mitigates some of these concerns."*(Discussion section)*
Study size	10	Explain how the study size was arrived at	Page 5, Paragraph 2;	Given the exploratory retrospective study design of the present study, no a priori sample size calculation was performed. Instead, a post hoc power analysis was conducted to evaluate the study’s ability to detect the primary effect of interest.

Continued on next page

Quantitative variables	11	Explain how quantitative variables were handled in the analyses. If applicable, describe which groupings were chosen and why	Page 5, Paragraph 1;	Categorical variables were reported as numbers (expressed as percentages) and analyzed with the χ^2 test.
Statistical methods	12	(*a*) Describe all statistical methods, including those used to control for confounding	Page 5, Paragraph 1	Normally distributed continuous variables were presented as mean ± standard deviation (x ± s) and compared using the independent samples t-test. Non-normally distributed variables were expressed as median (interquartile range) [M (Q1, Q3)] and compared using the Mann-Whitney U test. Categorical variables were reported as numbers (expressed as percentages) and analyzed with the χ^2 test. Binary logistic regression was performed to examine the association between clinical factors and complications. Factors with a p-value < 0.1 were included in the multivariate regression analysis, followed by collinearity diagnosis with a variance inflation factor (VIF) threshold set at < 5. The Box-Tidwell test was used to assess the linearity assumption between continuous variables and logit(P), and the results were validated via Bonferroni correction. The Hosmer-Lemeshow test was performed to

			assess the calibration performance of the model, where a p-value > 0.05 was indicative of adequate calibration. The model’s discriminative ability was evaluated by computing the area under the curve (AUC) and its 95% confidence interval (95% CI). Additionally, the Nagelkerke R^2 was reported to quantify the model’s goodness of fit. To assess the robustness of the model, a sensitivity analysis adjusting for confounding factors was performed. Data analyses were conducted using IBM SPSS Statistics (version 27.0). A two-tailed p-value < 0.05 was considered statistically significant.
	(*b*) Describe any methods used to examine subgroups and interactions	Page 5, Paragraph 1; Page 6, Paragraph 3;	To assess the robustness of the model, a sensitivity analysis adjusting for confounding factors was performed. Interaction term analysis and stratified analysis of surgical approaches were performed.
	(*c*) Explain how missing data were addressed	NA	This study employed a complete case analysis approach
	(*d*) *Cohort study*—If applicable, explain how loss to follow-up was addressed<br>*Case-control study*—If applicable, explain how matching of cases and controls was addressed<br>*Cross-sectional study*—If applicable, describe analytical methods taking account of sampling strategy	NA	This study employed a complete case analysis approach

		(*e*) Describe any sensitivity analyses	Page 5, Paragraph 1; Page 6, Paragraph 4;	To assess the robustness of the model, a sensitivity analysis adjusting for confounding factors was performed. Sensitivity analysis section: Alternative Modeling of SMI; Robustness Testing for VATI and PNI; Adjustment for key confounding factors
**Results**				
Participants	13*	(a) Report numbers of individuals at each stage of study—eg numbers potentially eligible, examined for eligibility, confirmed eligible, included in the study, completing follow-up, and analysed	Page 10, Paragraph 1; Page 11, Paragraph 2.	Figure 1, Table1.
		(b) Give reasons for non-participation at each stage	Page 10, Paragraph 1	Figure 1
		(c) Consider use of a flow diagram	Page 10, Paragraph 1	Figure 1
Descriptive data	14*	(a) Give characteristics of study participants (eg demographic, clinical, social) and information on exposures and potential confounders	Page 5, Paragraph 3	A total of 223 patients were included, with a median age of 50 years. Specifically, 101 patients (45.3%) were classified as FIGO 2018 stage I, and 122 patients (54.7%) as stage II. Squamous cell carcinoma was the predominant pathological type (83%). Open surgery was performed in 190 patients (85.2%), and 162 (72.6%) received postoperative adjuvant therapy. Regarding nutritional status, only 4 patients (1.8%) were

				underweight, where as 89 patients (39.9%) were overweight (Table1).
		(b) Indicate number of participants with missing data for each variable of interest	NA	This study employed a complete case analysis approach
		(c) *Cohort study*—Summarise follow-up time (eg, average and total amount)	Page 3, Paragraph 7	Complications occurring within 3 months postoperatively were analyzed based on patients' medical records and follow-up data.
Outcome data	15*	*Cohort study*—Report numbers of outcome events or summary measures over time	Page 3, Paragraph 7	Complications occurring within 3 months postoperatively were analyzed based on patients' medical records and follow-up data.
		Case-control study—Report numbers in each exposure category, or summary measures of exposure		
		Cross-sectional study—Report numbers of outcome events or summary measures		
Main results	16	(*a*) Give unadjusted estimates and, if applicable, confounder-adjusted estimates and their precision (eg, 95% confidence interval). Make clear which confounders were adjusted for and why they were included		
		(*b*) Report category boundaries when continuous variables were categorized	Page 4, Paragraph 4	Sarcopenia was defined using internationally widely adopted sex-specific cutoff values: for males, SMI< 43.0 cm^2/m^2 (when body mass index [BMI] $\geq$ 25 kg/m^2) or < 53.0 cm^2/m^2 (when BMI < 25 kg/m^2); for females, SMI < 41.0 cm^2/m^2.
		(*c*) If relevant, consider translating estimates of relative risk into absolute risk for a meaningful time period	Page 5, Paragraph 1; Page 6, Paragraph 2;	Variables with a P-value < 0.1 in the univariate analysis and those of established clinical relevance (age, body mass index, ASA classification, tumor stage, and surgical approach) were included in

				the multivariable logistic regression model to isolate the independent effect of body composition metrics by controlling for these potential confounders. *(Methods, 'Statistical Analysis' section)*<br>After adjustment for the pre-specified confounders (age, BMI, ASA score, tumor stage, and surgical approach) in the multivariate model, SMI (adjusted OR = 0.90, 95% CI: 0.85–0.96), VATI (adjusted OR = 1.04, 95% CI: 1.01–1.07), and PNI (adjusted OR = 0.92, 95% CI: 0.87–0.97) remained independent predictors. *(Results, 'Risk Factors for Postoperative Complications' section)*
Other analyses	17	Report other analyses done—eg analyses of subgroups and interactions, and sensitivity analyses	Page 14, Paragraph 1	Table5.
**Discussion**				
Key results	18	Summarise key results with reference to study objectives	Page 7, Paragraph 1	Previous studies on surgical complications in cervical cancer patients have primarily focused on clinicopathological factors and surgical approaches. However, the potential impact of body composition on postoperative complications in early-stage

				cervical cancer has not been previously reported. Our study demonstrates that sarcopenia, high VATI, and low PNI are significantly associated with an increased complication risk. These findings suggest that patients with favorable body composition profiles experienced fewer postoperative complications, highlighting the potential clinical benefits of interventions aimed at improving body composition.
Limitations	19	Discuss limitations of the study, taking into account sources of potential bias or imprecision. Discuss both direction and magnitude of any potential bias	Page 9, Paragraph 2	This study has several limitations. First, as a single-center retrospective analysis, it is susceptible to information bias. Second, the sample size of the laparoscopic surgery subgroup is relatively small, which may have limited statistical power in stratified analyses and potentially reduced the generalizability of the findings. Third, while multiple confounding factors were adjusted for, the possibility of residual confounding cannot be entirely ruled out.
Interpretation	20	Give a cautious overall interpretation of results considering objectives, limitations, multiplicity of analyses, results from similar studies, and other relevant evidence	Page 9-11	**Link to objective:** Our findings address the primary objective, identifying SMI and VATI as independent predictors of complications. (Discussion, Para 1)

				**Comparison with other studies:** Our results align with studies in colorectal cancer but contrast with those in ovarian cancer. (Discussion, Para 2-5)<br>**Limitations:** The retrospective design precludes causal inference, and residual confounding remains possible. (Discussion, Para 7)<br>**Multiplicity of analyses:** The consistent findings across multiple sensitivity analyses strengthen the robustness of our conclusions. (Discussion, Para 6)<br>**Cautious interpretation:** We suggest these metrics may help identify high-risk patients, but prospective validation is required. (Discussion, Para 6)
Generalisability	21	Discuss the generalisability (external validity) of the study results	Page 9, Paragraph 1-2	While the single-center design may limit generalisability, the use of objective body composition measures and alignment of our findings with broader oncological literature support the applicability of our results to similar patient populations in tertiary care settings.
**Other information**				
Funding	22	Give the source of funding and the role of the funders for the present study and, if applicable, for the original study on which the present article is based	Page 9, Paragraph 4	This study received no funding support.

*Give information separately for cases and controls in case-control studies and, if applicable, for exposed and unexposed groups in cohort and cross-sectional studies.

Note: An Explanation and Elaboration article discusses each checklist item and gives methodological background and published examples of transparent reporting. The STROBE checklist is best used in conjunction with this article (freely available on the Web sites of PLoS Medicine at http://www.plosmedicine.org/, Annals of Internal Medicine at http://www.annals.org/, and Epidemiology at http://www.epidem.com/). Information on the STROBE Initiative is available at www.strobe-statement.org.
