# Supplementary material for: The impact of body composition and systemic inflammatory markers on postoperative complications in early-stage cervical cancer
Source: Front Oncol. 2026 Jan 6;15:1696383. doi: 10.3389/fonc.2025.1696383 (PMC12815836; doi:10.3389/fonc.2025.1696383)
Supplement: Supplementary file 2 [file DataSheet2.pdf]

Supplementary Table 1: Exploratory Analysis Stratified by Surgical Approach (n = 223)

Variable	≥Grade 2 postoperative complications		
	Univariable		
	OR	95%CI	P
Laparoscopy(n=37)			
SMI (Non-sarcopenia vs sarcopenia)	0.36	0.16-2.05	0.253
VATI	1.01	0.96-1.06	0.741
Laparotomy(n=186)			
SMI (Non-sarcopenia vs sarcopenia)	0.49	0.27-0.89	0.018
VATI	1.03	1.01-1.04	0.004

Supplementary Table 2: Multivariate Analysis Excluding Patients Who Received Postoperative Blood Transfusion (n = 201)

Variable	≥Grade 2 postoperative complications		
	Multivariable		
	OR	95%CI	P
Tumor size(> 4cm vs≤ 4cm)	1.24	0.55-2.78	0.600
Surgical method (Laparoscopy vs Laparotomy)	0.43	0.17-1.12	0.084
Intraoperative blood loss (per 100 mL)	0.98	0.88-1.09	0.688
Postoperative Alb (Per 1 g/L)	0.95	0.86-1.05	0.329
SMI (Non-sarcopenia vs sarcopenia)	0.37	0.19-0.73	0.004
VATI (Per 1 cm2/m2)	1.04	1.02-1.06	0.001
PNI	0.91	0.84-0.99	0.019

Supplementary Table 3. Results of the Box-Tidwell Test for the Linear Assumption of Continuous Variables

Continuous variable	Interaction Term	Uncorrected P - value	P-value After Bonferroni Correction	Conclusion
IBL	IBL×Ln(IBL)	0.118	P < 0.0056	Supports the Linear Assumption
PAlb	PAlb×Ln(PAlb)	0.041	P < 0.0056	
VATI	VATI×Ln(VATI)	0.048	P < 0.0056	
PNI	PNI×Ln(PNI)	0.168	P < 0.0056	

IBL： Intraoperative blood loss (per 100 mL)； PAlb: Postoperative Alb；
